# Inhibition of the proteasome and proteaphagy enhances apoptosis in FLT3‐ITD‐driven acute myeloid leukemia

**DOI:** 10.1002/2211-5463.12950

**Published:** 2020-11-24

**Authors:** Rosa G. Lopez‐Reyes, Grégoire Quinet, Maria Gonzalez‐Santamarta, Clément Larrue, Jean‐Emmanuel Sarry, Manuel S. Rodriguez

**Affiliations:** ^1^ Institute of Advanced Technology and Life Sciences (ITAV) IPBS‐Centre de la Recherche Scientifique (CNRS) Université Toulouse III Paul Sabatier Toulouse France; ^2^ Cancer Research Center of Toulouse Unité Mixte de Recherche (UMR) 1037 INSERM ERL 5294 Centre de la Recherche Scientifique (CNRS) Toulouse France

**Keywords:** AML, bortezomib, FLT3‐ITD, leukaemia, proteaphagy, ubiquitin

## Abstract

Acute myeloid leukaemia (AML) is a clonal disorder that affects hematopoietic stem cells or myeloid progenitors. One of the most common mutations that results in AML occurs in the gene encoding fms‐like tyrosine kinase 3 (*FLT3*). Previous studies have demonstrated that AML cells expressing FLT3‐internal tandem duplication (ITD) are more sensitive to the proteasome inhibitor bortezomib (Bz) than FLT3 wild‐type cells, with this cytotoxicity being mediated by autophagy (Atg). Here, we show that proteasome inhibition with Bz results in modest but consistent proteaphagy in MOLM‐14 leukemic cells expressing the FLT3‐ITD mutation, but not in OCI‐AML3 leukemic cells with wild‐type FLT3. Chemical inhibition of Atg with bafilomycin A simultaneously blocked proteaphagy and resulted in the accumulation of the p62 Atg receptor in Bz‐treated MOLM‐14 cells. The use of ubiquitin traps revealed that ubiquitin plays an important role in proteasome‐Atg cross‐talk. The p62 inhibitor verteporfin blocked proteaphagy and, importantly, resulted in accumulation of high molecular weight forms of p62 and FLT3‐ITD in Bz‐treated MOLM‐14 cells. Both Atg inhibitors enhanced Bz‐induced apoptosis in FLT3‐ITD‐driven leukemic cells, highlighting the therapeutic potential of these treatments.

## Graphical abstract

Autophagy (Atg) regulates cytotoxicity after proteasome inhibition in acute myeloid leukaemia (AML) cells expressing fms‐like tyrosine kinase 3 (FLT3) mutations. Here, we show that the proteasome inhibitor bortezomib activates proteaphagy in AML cells expressing FLT3‐internal tandem duplication (ITD), but not in cells with wild‐type FLT3. Chemical inhibition of Atg blocking autophagosome‐lysosome fusion or the Atg receptor p62 efficiently inhibits proteaphagy and enhances bortezomib‐induced apoptosis in FLT3‐ITD‐driven leukemic cells.

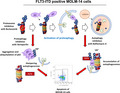

**Abbreviations**
ALS: autophagy‐lysosome system
AML: acute myeloid leukaemia
Atg: autophagy
BafA: bafilomycin A
Bz: bortezomib
FLT3: fms‐like tyrosine kinase 3
FT: flow‐through
GST: glutathione S‐transferase
IP: immunoprecipitation
ITD: internal tandem duplication
LC3B: microtubule‐associated proteins 1A/1B light chain 3B
PI: proteasome inhibitor
TUBEs: tandem ubiquitin binding entities
UPS: ubiquitin‐proteasome system
VT: verteporfin

Acute myeloid leukaemia (AML) is a clonal disorder that affects hematopoietic stem cells or myeloid progenitors. It is characterized by an accumulation of immature leukemic cells in the bone marrow and peripheral blood and leads to bone marrow failure [1]. AML is a heterogeneous disease with a variety of distinct genetic alterations [2]. One of the most common mutations occurs in the gene encoding fms‐like tyrosine kinase 3 (*FLT3*) [3]. This type III receptor tyrosine kinase regulates the normal growth and differentiation of hematopoietic cells via the activation of multiple signalling including Akt, mitogen‐activated protein kinase and signal transducer and activator of transcription 5 (STAT5) [4, 5]. FLT3 cooperates with other recurrent molecular abnormalities to induce acute leukaemia in preclinical models. Internal tandem duplication (ITD) mutations in the FLT3 gene are found in approximately 30% of AML patients and are associated with a poor clinical outcome. Previous studies have demonstrated that AML cells expressing FLT3‐ITD are more sensitive to the proteasome inhibitor (PI) bortezomib (Bz) than FLT3 wild‐type cells. This cytotoxicity is mediated by autophagy (Atg) [6]. Furthermore, the genetic inhibition of early Atg steps or of autophagosome formation blocks FLT3‐ITD, STAT5 and Akt degradation induced by Bz [6].

The proteasome has been envisioned as a promising target for the development of anticancer therapeutic drugs [7]. The 26S proteasome is a large multi‐subunit protease (1500–2000 kDa) formed by the 20S proteolytic core and one or two 19S regulatory particles [8]. Three proteolytically active subunits integrate the 20S core: β1 with a caspase‐like activity, β2 with a trypsin‐like activity and β5 with a chymotrypsin‐like activity. β5 is the primary target for most PIs that reached a clinical phase [8, 9]. Cancer therapy also targets the autophagy‐lysosome system (ALS), another proteolytic process that is responsible for the bulk degradation of cytoplasmic components. Amino acid deficiency activates Atg by regulating the signalling cascades controlling this proteolytic pathway [10]. During selective Atg, phagophores engulf cytoplasmic material, and then fuse to form double‐membrane autophagosomes. Cargo recruitment occurs through a family of Atg receptors, including p62, OPTN or NBR1, which are often used as markers for Atg activation together with the ubiquitin‐like molecule Atg8 [11]. The Atg machinery was first identified in yeast and equivalent molecules reported in mammalian cells [11, 12, 13]. Distinct Atg events drive the degradation of organelles or aggregated proteins such as mitophagy (mitochondria), aggrephagy (protein aggregates) or proteasome (proteaphagy), to name a few [14].

Many of the current Atg inhibitors act at a late stage of the system, such as the V‐ATPase inhibitor bafilomycin A (BafA). BafA inhibits Atg in a non‐selective way, by neutralizing the acidic pH of the lysosomal hydrolases that drive autophagic degradation [15]. Verteporfin (VT) is a drug approved by the US Food and Drug Administration that was identified in a screen for chemicals that prevent autophagosome formation [16]. Unlike BafA, VT inhibits Atg at an early stage and does not allow autophagosome accumulation[16]. A better understanding of the proteolytic regulation mechanism and interplay will allow the exploration of alternative/combined treatments to tackle cancer development and/or drug resistance.

Here, we aimed to better understand the proteolytic cross‐talk connecting proteasome with Atg after Bz treatment in FLT3‐ITD‐positive MOLM‐14 AML cells. Using a chemical approach to block Atg at distinct levels together with ubiquitin traps [known as tandem ubiquitin binding entities (TUBEs)] [17], immunoprecipitation (IP) and immunofluorescence, we found that proteaphagy was activated after Bz treatment. Although proteaphagy is a process preserved in distinct species [18, 19, 20], we found that the presence of FLT3‐ITD predisposes MOLM‐14 cells to activate it.

## Materials and methods

### Cell lines

Human myeloid leukaemia cell lines MOLM‐14 and OCI‐AML‐3 were purchased from the ATCC collection (ATCC, Manassas, VA, USA). AML cell lines were maintained in RPMI supplemented with 10% foetal calf serum in the presence of 100 U·mL^−1^ penicillin and 100 µg·mL^−1^ streptomycin. Cells were incubated at 37 °C with 5% CO_2_ [6]_._ To facilitate Atg analysis, 2% calf serum was used during the chemical inhibitor treatment for a maximum of 8 h.

### Antibodies and reagents

The anti‐microtubule‐associated protein antibodies 1A/1B light chain 3B (LC3B), anti‐Erk 1/2, anti‐STAT5, anti‐Akt, anti‐ubiquitin (P4D1) and anti‐PSMB5 were purchased from Cell Signaling Technology (Beverly, MA, USA). Anti‐FLT3, anti‐p62/SQSTM1 and anti‐Beta 2 were obtained from Santa Cruz Biotechnology (Dallas, TX, USA) and anti‐PSMA6 was obtained from Invitrogen (Carlsbad, CA, USA). Anti‐PSMB6 and anti‐Rpn10 were purchased from Enzo (Enzo Diagnostics, Farmingdale, NY, USA), anti‐PSMD3 was obtained from Thermo Fisher (Waltham, MA, USA) and anti‐GAPDH was obtained from Sigma‐Aldrich (St Louis, MO, USA).

Bz and chloroquine were purchased from Sigma‐Aldrich, BafA was obtained from InvivoGen (InvivoGen, San Diego, CA, USA) and VT was obtained from Sigma‐Aldrich.

### Western blot analysis

Proteins were resolved using 8–12% PAGE and electro‐transferred to poly(vinylidene difluoride) membranes. Membranes were then blocked with 5% skimmed powdered milk in NaCl/Tris or 5% BSA in NaCl/Tris. Membranes were immunostained with appropriate antibodies and horseradish peroxidase‐conjugated secondary antibodies and visualized using an enhanced chemiluminescence system.

### Apoptosis assay

Cell lines were cultured in RPMI 5% and then treated at different times with Bz, BafA and VT. Then, 5 × 10^5^ cells were washed with NaCl/P_i_ and resuspended in 100 µL of annexin‐V binding buffer. Two microliters of annexin‐V‐fluorescein isothiocyanate was added at room temperature. All samples were analysed by a fluorescence‐activated cell sorter (FACS Calibur flow cytometer; Becton‐Dickinson Biosciences, Franklin Lakes, NJ, USA) [6].

### TUBE capture

TUBEs were produced as described by Hjerpe *et al*. [17, 21]. Twenty million cells were used for each condition [TUBE p62, TUBE HHR23 or glutathione *S*‐transferase (GST) control]. Cells were resuspended in 500 µL of TUBE lysis buffer, maintained for 10 min at 4 °C and spin down at 15 500 ***g***. A fraction of this supernatant was diluted in 3 × boiling buffer (BB) (50 mm Tris‐HCl, pH 6.8, 10% glycerol, 2% SDS, bromophenol blue, 10% β‐mercaptoethanol) and considered as the input. Supernatant was transferred to the glutathione‐agarose beads with TUBEs or GST control, and samples were incubated overnight in rotation at 4 °C. The next day, samples were spin down at 500 ***g*** at 4 °C, a fraction recollected, diluted in BB and considered as the flow‐through (FT). Beads were washed three times with 1 mL of NaCl/P_i_ Tween 0.05%. Beads were resuspended in 100 µL of BB and boiled for 5 min before protein electrophoresis.

### Antibody cross‐linking

Thirty microliters per point of magnetic beads protein A (Millipore, Billerica, MA, USA) were washed with NaCl/P_i_ and equilibrated in binding buffer (50 mm Tris pH 8.5, 150 mm NaCl, +0.5% NP40) using a magnetic holder. Antibodies were added to beads and rotated overnight. The next day, 10–20 µL of supernatant was kept to control antibody binding. Beads were washed twice with NaCl/P_i_ and once with 500 µL of coupling buffer (200 mm borate, 3 m NaCl pH 9). Fifty millimolars of dimethyl pimelimidate was added to the coupling buffer, and samples were rotated for 30 min with this cross‐linking solution. Supernatant was discarded, replaced with fresh cross‐linking solution and incubated at 4 °C for 30 min. Beads were washed twice with coupling buffer before blocking with 20 mm ethanolamine, pH 8.2. Supernatant was discarded and replaced by fresh ethanolamine and incubated for 1 h. Beads were washed twice with NaCl/P_i_. Non‐coupled antibodies were removed with two washes of 1 m NaCl/binding buffer. A NaCl/P_i_ equilibration was performed before washing three times with 200 mm glycine, pH 2.5. Beads were blocked with 0.1% BSA in binding buffer for 90 min. Magnetic beads were equilibrated in binding buffer and maintained in NaCl/P_i_ until use. A fraction of these beads (10–20 µL) were analyzed by electrophoresis followed by Coomassie blue staining to compare antibodies before and after cross‐linking.

### IP in the presence of TUBEs

Twenty millions of cells were spin down at 300 ***g*** for 10 min and the dry pellet was resuspended in 500 μL of TUBE lysis buffer including 100 μg of TUBE p62 or TUBE HHR23[17]. Cell lysates were homogenized with 40 strokes at 4 °C using a Dounce homogenizer. The whole sample was centrifuged at 200 ***g*** for 5 min and the supernatant was recovered for IP. A fraction (1/20) of the supernatant was considered as input. The cross‐linked antibody was incubated with cell lysates in rotation for 1 h 30 min at 4 °C. Samples were disposed in magnetic holder to separate bound from unbound material. Proteins unbound to cross‐linked antibodies were considered as the FT fraction. Magnetic beads were washed with NaCl/P_i_/Tween 0.05% three to five times and then resuspended in 30 µL of BB 3× to be analyzed by western blotting.

### Statistical analysis

Data from four independent experiments are reported as the mean ± SEM. Statistical analyses were performed using unpaired two‐tailed Student’s *t‐*tests with prism, version 4 (GraphPad Software Inc., San Diego, CA, USA). *P* < 0.05 was considered statistically significant.

## Results

### Proteaphagy activation after proteasome inhibition in FLT3‐ITD mutant‐driven MOLM‐14 leukemic cells

**Fig. 1:**
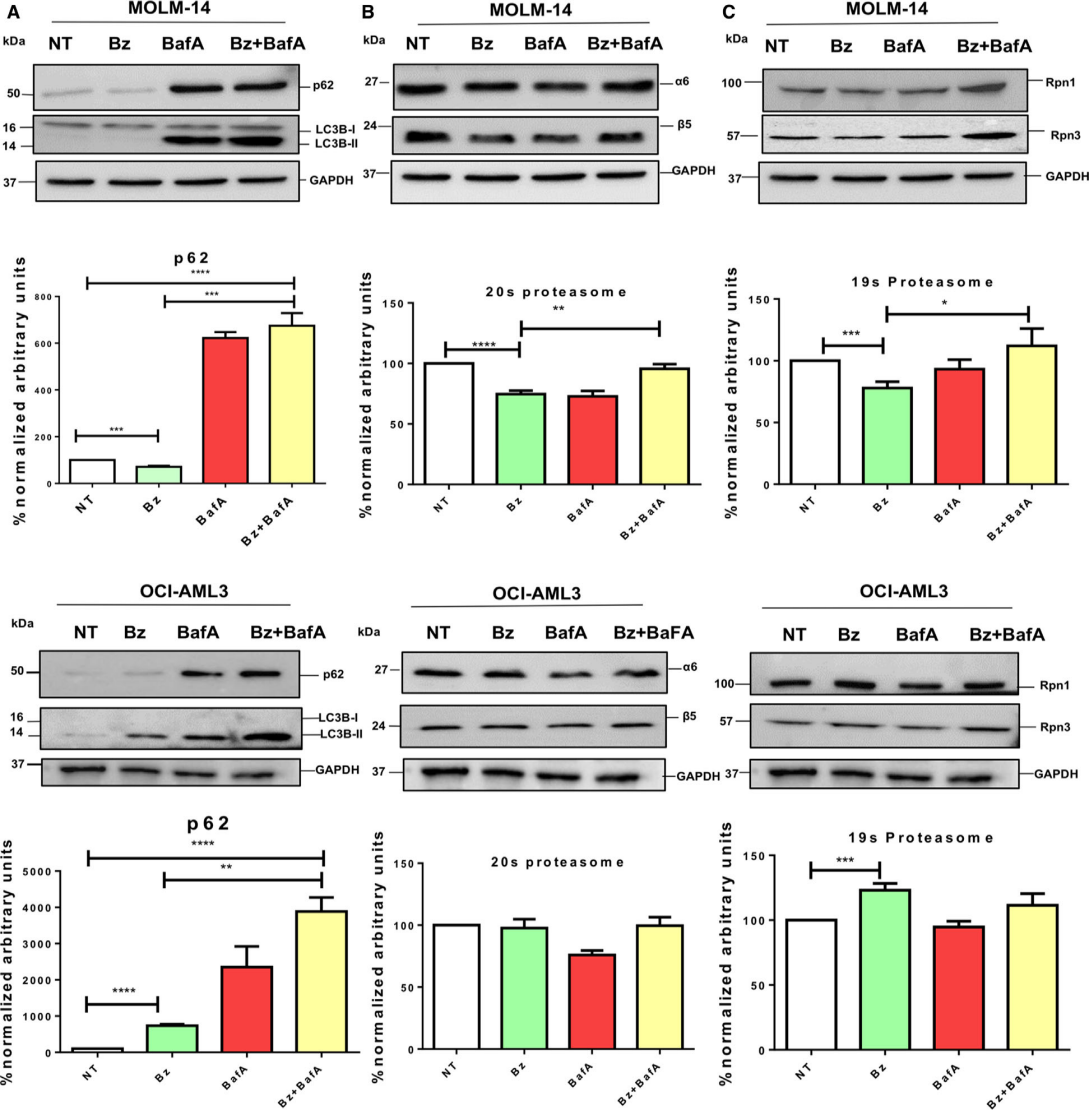
Bz‐driven proteaphagy is enhanced in the FLT3‐ITD phenotype. MOLM‐14 (FLT3‐ITD+/−) or OCI‐AML3 (FLT3‐WT) cells were treated for 8 h with 10 nm Bz and 20 nm bafilomycin. Total cell lysates were resolved by SDS/PAGE and immunoblotted with the indicated antibodies recognizing the Atg receptor p62 (A), proteasome core subunits α6 and β5 (B) or 19S subunits Rpn1 and Rpn3 (C). Protein expression levels were quantified by densitometry analysis (imagej; NIH, Bethesda, MD, USA). Statistical analyses were performed using unpaired two‐tailed Student’s *t‐*tests with prism, version 4. *P < 0.05, **P < 0.01, ***P < 0.001 and ****P < 0.0001. Data are reported as the mean ± SEM (*n* = 4).

**Fig. 2:**
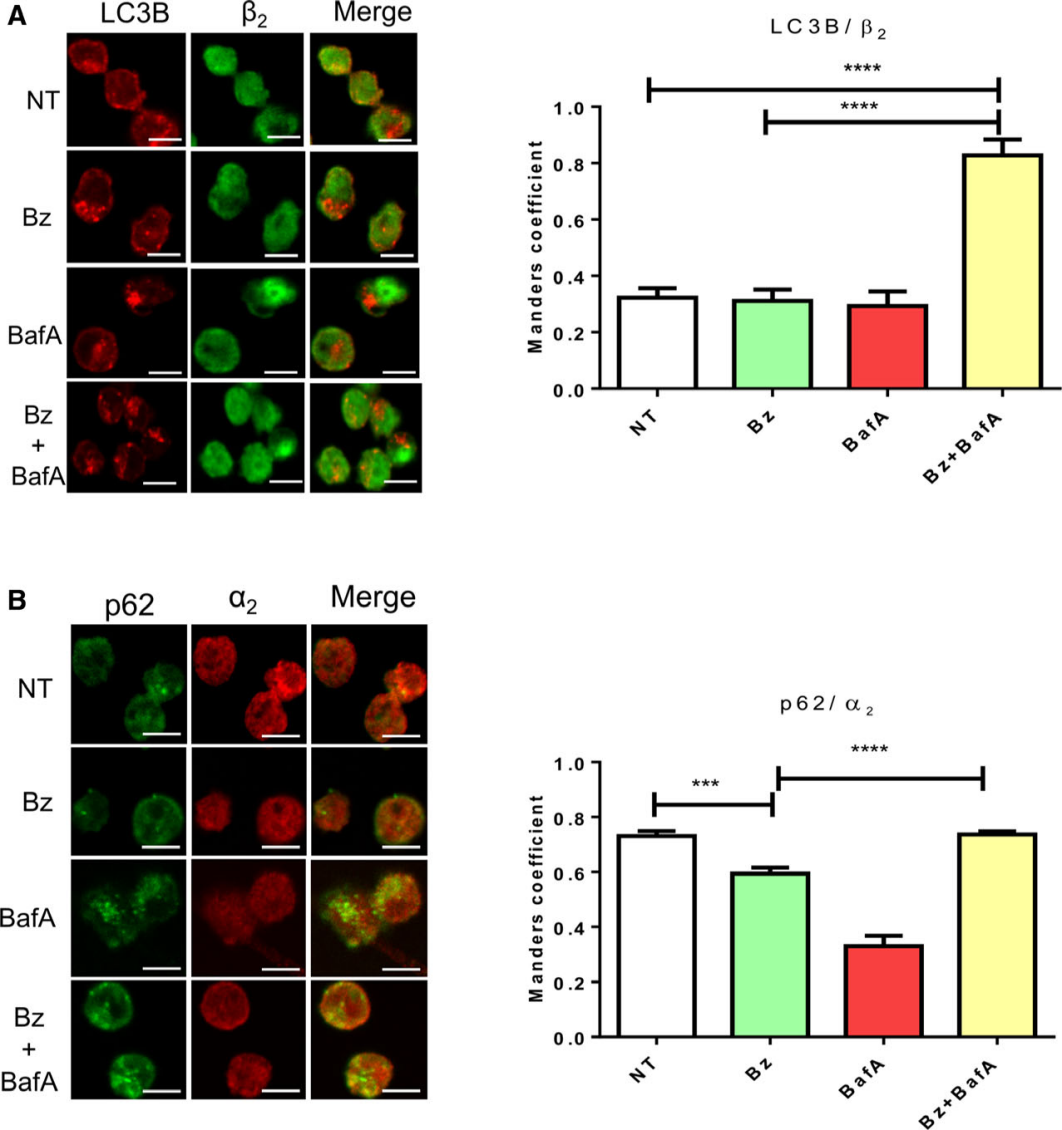
Colocalization of proteasome and Atg markers after Bz and Atg inhibitor treatment in FLT3‐ITD AML cells. Indirect immunofluorescence staining LC3B/β_2_ (A)‐ or p62/α_2_ (B)‐positive structures in MOLM‐14 cells treated for 8 h with 10 nm Bz and 20 nm BafA. Images were captured by confocal microscopy. Scale bar = 10 µm. Immunofluorescence images were quantified from three replicates. Statistical analyses were performed using unpaired two‐tailed Student’s *t*‐tests with prism, version 6. ***P < 0.001 and ****P < 0.0001. Data are reported as the mean ± SEM.

To mechanistically understand the regulation of the cross‐talk between the ubiquitin‐proteasome system (UPS) and ALS in leukaemia, we investigated the role of proteaphagy, which is known to be activated after proteasome inhibition [22]. The impact of Bz was assessed in MOLM‐14 cells with the FLT3‐ITD mutation and compared with the FLT3 wild‐type cell line OCI‐AML3 (Fig. 1). Bz treatment does not always promote an obvious degradation of proteasome subunits because proteolysis can be compensated by the *novo* synthesis of these subunits as reported previously [23, 24]. For this reason, proteaphagy was evaluated by the degradation of 20S and 19S proteasomal subunits after Bz treatment and their accumulation with Atg inhibitors. BafA treatment resulted in the accumulation of Atg markers p62 and LC3B in the presence or absence of Bz, indicating that Atg was activated under these experimental conditions in both cell lines (Fig. 1A). Nevertheless, lipidated forms of LC3B were only observed after BafA treatment in MOLM‐14 but not in OCI‐AML3. The low levels of apoptosis observed after 8 h of individual or combined Bz/BafA treatment excluded the possibility that differences could be due to massive death of MOLM‐14 cells (Fig. S1). Our results showed modest but consistent Bz‐mediated degradation of 20S proteasome subunits α6s and β5 and 19S subunits Rpn1 and Rpn3, which was blocked by BafA in MOLM‐14 (Fig. 1B,C). However, the combination of BafA with Bz did not significantly accumulate proteasome subunits in OCI‐AML3 as was the case in MOLM‐14 cells (Fig. 1B,C, lower), suggesting that a predisposition for degradation of 26S proteasome could be linked to the presence of FLT3‐ITD. These results were also confirmed by immunofluorescence, where proteasomes subunit β2 or α2 colocalized with autophagosomes (Atg8 equivalent LC3B or p62 staining, respectively) after Bz/BafA treatment of MOLM‐14 cells (Fig. 2). This Bz‐induced degradation of proteasome subunits is blocked by BafA, indicating that proteaphagy mediated these proteolytic events.

### Role of p62 and ubiquitin in Bz‐induced autophagy

**Fig. 3:**
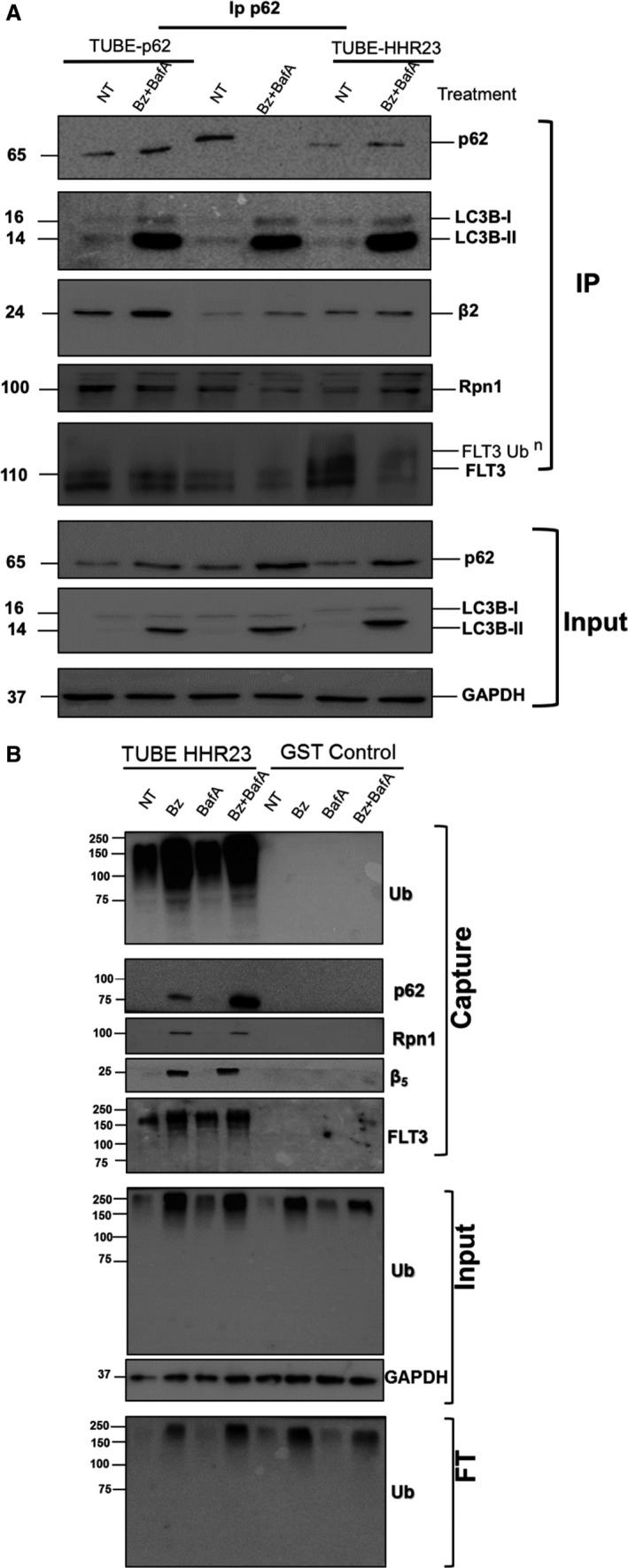
Ubiquitin role in proteaphagy and degradation of FLT3‐ITD under proteasome inhibition conditions. MOLM‐14 cells were treated (or not) for 8 h with 10 nm Bz, 20 nm BafA, or both drugs, and cells were lysed as reported [17]. Ubiquitylated proteins were captured using TUBEs‐HHR23, TUBEs‐p62 or GST control (A). Captured proteins were resolved by SDS/PAGE and immunoblotted with the indicated antibodies. Input and FT fractions were also analysed with anti‐ubiquitin antibody. GAPDH was used as the loading control. (B) MOLM‐14 cells were treated (or not) with Bz/BafA under the same conditions as in (A). p62‐bound proteins were captured by IP with a specific p62 antibody in the presence or absence of TUBE‐HHR23 or TUBE‐p62. Precipitated material was analysed by western blot with the indicated antibodies. The input fraction was analysed for the indicated proteins.

To analyse the role of p62 in the Bz‐induced autophagic degradation of the proteasomes and FLT3‐ITD, the interaction of p62 with proteasome subunits and FLT3‐ITD was investigated. IP experiments using a specific p62 antibody were performed using MOLM‐14 cells treated (or not) with Bz/BafA. Taking advantage of the protective effects of TUBEs that block the action of proteases, accumulate ubiquitylated proteins and interact with multiple chain types [17, 25], IPs were performed in the presence or absence of TUBEs based in the UBA domain of HHR23 (TUBE‐HHR23) or the UBA domain of p62 (TUBE‐p62) (Fig. 3A). In the absence of TUBEs, p62 was immunoprecipitated without treatment and Bz/BafA reduced the level of precipitated p62. However, in the presence of both TUBEs, p62 was protected from proteasome‐ and/or Atg‐mediated degradation under Bz/BafA conditions (Fig. 3A). Compared to the situation without any TUBEs or TUBE‐HHR23, the presence of TUBE‐p62 allowed a better coimmunoprecipitation of p62‐bound proteasome subunits β2 and but not RPN1. High molecular weight forms most likely representing ubiquitylated forms of RPN1 were better immunoprecipitated in the presence of TUBE‐HHR23 (Fig. 3A). Consistent with these observations, FLT3‐ITD was also protected by TUBE‐p62, although putative ubiquitylated forms of this protein were better immunoprecipitated in the presence of TUBE‐HHR23.

Because ubiquitin is a major coordinator of UPS and ALS, we investigated its role in the cross‐talk of these pathways induced after Bz and BafA treatments. In particular, we were interested in investigating whether the high molecular weight forms of FLT3 co‐immunoprecipitated with p62 corresponded to ubiquitylated FLT3. TUBE‐HHR23 was used to capture total ubiquitylated proteins from MOLM‐14 cells treated (or not) with individual and combined Bz/BafA treatment (Fig. 3B). Total ubiquitylated proteins were efficiently trapped by TUBE‐HHR23. BafA treatment alone did not significantly enrich ubiquitylated proteins captured by TUBE‐HHR23 compared to Bz or Bz/BafA. Proteasome subunits β5 and Rpn1 were captured by TUBE‐HHR23 (Fig. 3B). The p62 receptor was also captured under the same conditions, although the combination of Bz/BafA enriched this protein compared to Bz alone (Fig. 3B). Interestingly, ubiquitylated forms of FLT3‐ITD were captured by TUBE‐HHR23 under all conditions but were best enriched when Bz/BafA treatment was used (Fig. 3B).

### p62 drives proteaphagy and autophagic degradation of FLT3‐ITD

**Fig. 4:**
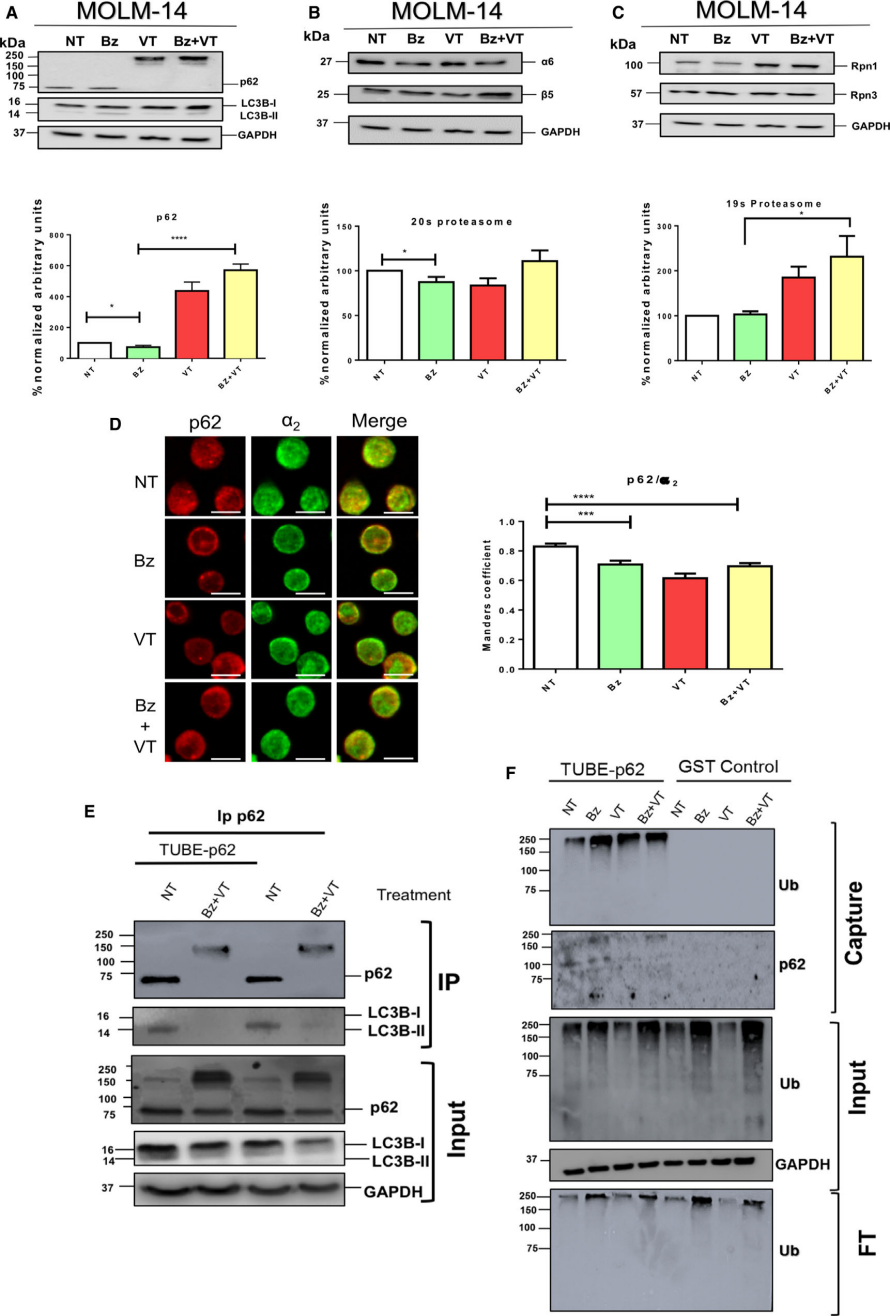
p62 drives proteaphagy in FLT3‐ITD AML cells. MOLM‐14 cells were treated for 8 h with 10 nm Bz and 1 µm VT. Total cell lysates were resolved by SDS/PAGE and immunoblotted with antibodies against Atg markers LCB3 and p62 (A), proteasome core subunits α6 and β5 (B), and 19 subunits Rpn1 and Rpn3 (C). Protein expression levels were quantified by densitometry analysis (imagej). (D) Indirect immunofluorescence staining of p62/α_2_‐positive structures in MOLM‐14 cells treated for 8 h with 10 nm Bz and 1 µM VT. Images were captured by confocal microscopy. Scale bar = 10 µm. Immunofluorescence images were quantified from three replicates. Statistical analyses were performed using unpaired two‐tailed Student’s *t‐*tests with prism, version 6. *P < 0.05, **P < 0.01, ***P < 0.001 and ****P < 0.0001. Data are reported as the mean ± SEM (*n* = 5). (E) IP of p62 from MOLM‐14 cells treated (or not) with 10 nm Bz and 1 µm VT. Experiments were performed in the presence or absence of TUBE‐p62. Precipitated material was analysed by western blotting with specific p62 and LC3B antibodies. The input fraction was analysed with the indicated antibodies. (F) Capture of ubiquitylated proteins using TUBE‐p62 trap. GST was used as the negative control. Captured proteins were analysed by western blotting with anti‐ubiquitin or p62 antibodies. The input and FT fractions were analysed with the indicated antibodies.

To further assess the role of the Atg receptor p62 in proteaphagy, VT was used to treat MOLM‐14 cells and western blot analyses were performed to detect distinct proteasome subunits. High molecular weight forms of p62 were detected after VT treatment (Fig. 4A). Interestingly, the lipidated form of LC3B decreased after VT treatment, indicating that this drug reduced the Atg flux. Bz‐mediated degradation of 20S or 19S subunits was blocked by VT even if the Bz‐mediated degradation of 19S subunits was more prominent than 20S subunits (Fig. 4B,C). The colocalization of p62/α_2_ was significantly reduced with VT or Bz/VT, indicating that blocking of p62 could inhibit proteaphagy via a mechanism distinct from BafA (Fig. 4D). To investigate whether the high molecular weight forms of p62 observed in Fig 4A were aggregated and/or ubiquitylated, IP was performed with p62 antibodies in the presence or absence of TUBE‐p62 (Fig 4E) and TUBE capture of ubiquitylated proteins (Fig. 4F). Interestingly, the aggregated forms of p62 generated after Bz/VT treatment did not interact with the lipidated form of LC3B, indicating that this treatment negatively affected the interaction (Fig. 4E). The lipidated forms of LCB3 were also reduced in the input fraction, indicating that VT stops the Atg flux as observed in Fig. 4A. To explore the ubiquitylation status of p62 after VT treatment, we captured ubiquitylated proteins using TUBE‐p62 (Fig. 4F). VT had a negative impact on the total ubiquitylated proteins as can be observed in the input fraction. Nevertheless, the levels of ubiquitylation could be recovered when VT was combined with Bz. These results were also observed with respect to the TUBE capture of total ubiquitylated proteins. Under these experimental conditions, p62 increased its ubiquitylated levels after Bz treatment and decreased when cells were treated with VT (Fig. 4F). Taken together, the evidence indicates that, after VT treatment, aggregated forms of p62 are accumulated and these forms are less ubiquitylated. The reduction of the interaction p62/lipidated LC3B indicates that this p62 was not integrated into autophagosomes after Bz/VT treatment. In a similar manner, VT reduced the localization of p62 with proteasome subunits, supporting the idea that proteaphagy is hampered by this treatment (Fig. 4D). Thus, these data show that p62 plays an important role in the proteaphagy observed in MOLM‐14 cells.

### Inhibition of UPS and ALS pathways enhances apoptosis of FLT3‐ITD cells

**Fig. 5:**
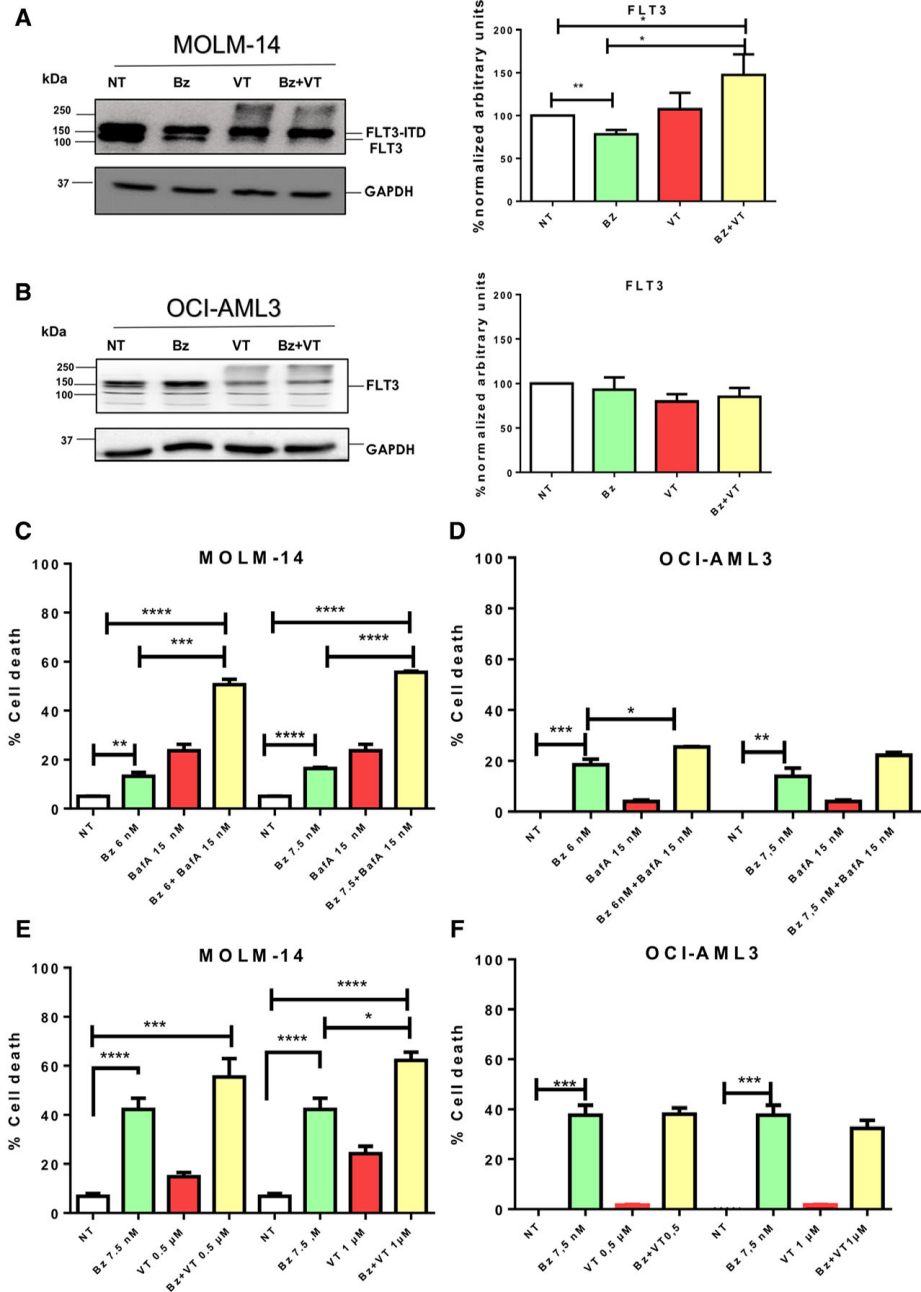
Proteasome and Atg inhibitors cooperate to improve apoptosis of FLT3‐ITD expressing cells. MOLM‐14 (A) and OCI‐AML3 (B) cells were treated for 8 h with 10 nm Bz and 1 µm VT. Total cell lysates were resolved by SDS/PAGE and immunoblotted with antibodies against FLT3 (A, B). Protein expression levels were quantified by densitometry analysis (imagej). Statistical analyses were performed using unpaired two‐tailed Student’s *t*‐tests tests with prism, version 6. *P < 0.05, **P < 0.01, ***P < 0.001 and ****P < 0.0001. Data are reported as the mean ± SEM. (C, D) MOLM‐14 and OCI‐AML3 cells were treated for 8 h with 15 nm BafA. Bz was added at a concentration of 6 or 7.5 nm for an additional 16 h. (E, F) MOLM‐14 and OCI‐AML3 cells were treated with a fixed concentration of 7.5 nm Bz and two distinct doses of VT (0.5 and 1 µm) as indicated. Apoptosis was analysed by FACS. The percentage of cell death was measured from four biological replicates. Statistical analyses were performed using unpaired two‐tailed Student’s *t‐*tests prism, version 4. *P < 0.05, **P < 0.01, ***P < 0.001 and ****P < 0.0001. Data are reported as the mean ± SEM (*n* = 3).

Individual or combined treatments were used to investigate whether Bz‐induced degradation of FLT3‐ITD was blocked by VT in MOLM‐14 cells (Fig. 5A). The results obtained indicated that FLT3‐ITD and FLT3 were degraded by up to 25% after Bz treatment in MOLM‐14 cells (Fig. 5A). FLT3‐ITD degradation was blocked by VT, whereas high molecular weight forms of FLT3 were formed under the same conditions in MOLM‐14 suggesting a ubiquitin‐driven Atg‐mediated proteolysis event (Fig. 5A). Interestingly, in OCI‐AML3 cells, under the same experimental conditions, FLT3 was not degraded by Bz and VT treatment and, instead, there was an accumulation of high mobility forms of this protein (Fig. 5B). The double Bz/VT treatment did not result in a significant accumulation of FLT3 in OCI‐AML3 (Fig. 5B), supporting the idea that proteolytic pathways are not efficiently activated in these cells.

**Fig. 6:**
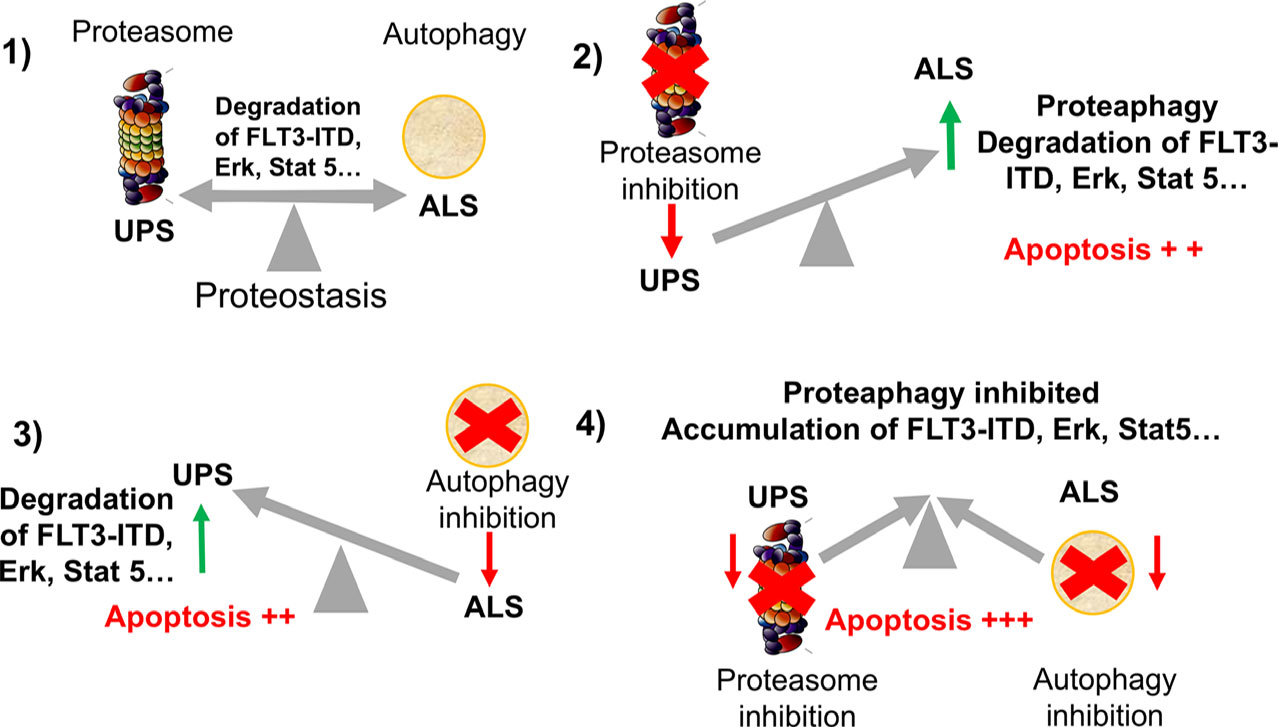
UPS and ALS cross‐talk under proteasome and/or Atg inhibition. (1) Under basal unstimulated conditions, turnover of important cellular factor is ensured by equilibrated proteolytic pathways. (2) Inhibition of proteasome directs crucial cellular factors to ALS for degradation. (3) Atg inhibition drives the degradation of some cellular factors to proteasome‐mediated degradation. (4) When both proteolytic pathways are impaired, both UPS and ALS contribute to accumulate cellular factors and increase apoptosis in FLT3‐ITD‐positive cells.

Finally, we investigated the consequences of accumulated proteasome and FLT3‐ITD after inhibition of the UPS and ALS pathways in MOLM‐14 cells compared to OCI‐AML3 cells. Apoptosis was measured by analysing annexin‐V‐positive cells after single agent or combined treatments (Fig. 5). Bz‐induced cell death was always maintained below 50% to differentiate positive or negative effects of BafA on the Bz treatments. To improve the efficiency of Atg inhibition, cells were pre‐treated with BafA for 8 h before adding Bz for an additional 16 h at the indicated doses (Fig. 5C,D). The results obtained showed that the toxicity of the individual BafA treatment was approximately 20% (MOLM‐14) or below 5% (OCI‐AML3) and both drugs efficiently cooperated to enhance the cell killing effect on MOLM‐14 (Fig. 5C), although this was not in the case in OCI‐AML3 (Fig. 5D). Apoptosis induced by VT also improved the results observed with Bz alone in MOLM‐14 cells (Fig. 5E) but not in OCI‐AML3 cells (Fig. 5F). In this case, Bz and VT were simultaneously added to cell cultures to work below the VT IC_50_. This results in an additional 8 h of Bz compared to Fig. 5A, explaining the higher apoptosis observed for the Bz treatment only. Cooperative effects observed with the double Bz/VT treatment were statistically significant in MOLM‐14 cells but not in OCI‐AML3 (Fig. 5E,F). Taken together, our results show that the inhibition of both proteolytic pathways markedly enhances apoptosis levels in MOLM‐14 that express FLT3‐ITD (Fig. 6) but not in OCI‐AML3 that express WT FLT3.

## Discussion

Multiple mechanisms of interplay between the UPS and ALS have been documented over the last 10 years. In the present study, we have identified proteaphagy as part of the selective autophagic events, which are activated after Bz treatment in FLT3‐ITD‐positive leukemic cells. Our data showed that this tyrosine kinase translocation facilitates the Bz‐mediated proteolysis of 20S and 19S subunits and their colocalization within autophagosomes. FLT3‐ITD can potentially predispose to proteaphagy as a result of its capacity to activate multiple signalling cascades that have an impact on Atg activation. Among these are the phosphatidylinositol‐3 kinase [26, 27]; Akt [28]; mammalian target of rapamycin [29]; Ras; and extracellular signal‐related kinase, mitogen‐activated protein kinase and STAT5 [5, 30] pathways. It remains to be determined whether any (or several) of these signalling pathways has a positive or negative impact on proteaphagy.

Proteaphagy is a complicated process to analyse because not all proteasomes are directly concerned by this type of degradation. Nuclear proteasomes will not be immediately affected because proteaphagy is a cytoplasmic event. According to the literature, only 20–50% of the proteasomes are regulated by proteaphagy depending on the time, intensity and type of stimuli in distinct biologic models [18, 19, 20]. Our results demonstrate that the Atg receptor p62 is implicated in the proteaphagy activated by Bz in MOLM‐14 cells. However, our data do not exclude the participation of other Atg receptors in this process [14]. For example, both the ubiquitin receptor Cue5 and the chaperon Hsp40 [19] or the proteasome subunit RPN10 [18], respectively, mediate proteaphagy in *Saccharomyces cerevisiae* or *Arabidopsis thaliana*. Nevertheless, the use of VT in leukemic cells showed that the inactivation of p62 stops Bz‐induced proteaphagy, supporting a major role for p62 in this process. Interestingly, VT favors the formation of high molecular weight aggregates of p62 [31], which are also observed with FLT3‐ITD with the same treatment. However, other effects have been reported for VT, including the activation of ROS, which could affect other processes [32, 33] making it difficult to attribute the effects of VT only to its action on p62.

The IP/protection assays [17] revealed that TUBE‐p62 protects p62, RPN1, β2 or FLT3‐ITD from Bz‐driven degradation blocked with BafA in MOLM14 cells. TUBE‐HHR23 also protects these factors, although to a lesser extent than TUBE‐p62. However, TUBE‐HHR23 better accumulates ubiquitylated forms of RPN1 or FLT3‐ITD. Similar IP experiments performed with Bz/VT in MOLM‐14 showed that high molecular weight forms of p62 did not interact with the lipidated form of LC3B, indicating that p62 was not integrated into autophagosomes under those conditions. This could be associated with the reduction of total ubiquitylated forms observed after VT treatment that might have a negative impact on ubiquitin‐regulated events.

The ubiquitin proteome captured with TUBE‐HHR23 includes several proteins implicated in proteaphagy such as p62, RPN1 or β5 after Bz or Bz/BafA treatments but not with BafA alone, indicating that this Atg inhibitor does not accumulate ubiquitylated forms of these factors in the absence of proteasome inhibition. TUBE‐p62 captures significantly less ubiquitylated proteins than TUBE‐HHR23 and specific ubiquitylated forms can only be seen with overexposure. Nonetheless, in this way, we found that, although Bz accumulated ubiquitylated forms of p62, VT reduces these forms, most likely by interfering with the integration of this receptor into autophagosomes. The use of both TUBEs to investigate UPS and ALS regulated events highlights the need to consider all possibilities with respect to determining which pathway plays a role under distinct experimental settings. Although TUBE‐HHR23 recognises almost all types of chains [17, 25, 34], the UBA domain of p62 recognises mainly K63 ubiquitin chains, explaining the observed differences [35, 36].

Taken together, our data indicate that, when both proteolytic pathways are blocked, the accumulation of cellular factors occurs as a result of the functional absence of these degradation machineries (Fig. 6). This contributes to enhanced apoptosis in MOLM‐14 but not in OCI‐AML3 cells under the same experimental conditions. In conclusion, targeting protein homeostasis could be an alternative for improving current treatments of FLT3‐ITD AML cells. Although the cross‐talk of these complex proteolytic mechanisms remains to be fully clarified, our results open new possibilities for the treatment of this AML phenotype.

## Conflict of interest

The authors declare no conflict of interest.

## Author contributions

RGLP, MSR and JES conceived and designed the project. RGLP, GQ and MGS acquired the data. RGLP, GQ, MGS and CL analysed and interpreted the data. MSR and RGLP wrote the pape. All authors approved the final

version of the manuscript submitted for publication.

## Supporting information


**Fig. S1.** Cell death evaluation at 8 h. Treatments using 15 nm Bz and 10 nm BafA with fetal bovine serum 2% MOLM‐14 before annexin‐V staining and flow cytometry to validate the western blot conditions (*n* = 24), three biological replicates. Statistical analyses were performed using unpaired two‐tailed Student’s t‐tests with prism, version 6. *P < 0.05, **P < 0.01, ***P < 0.001 and ****P < 0.0001. Data are reported as the mean ± SEM (*n* = 3).

## Data Availability

The data are available from the corresponding author upon reasonable request.
