# Supplementary figures and images for: Inhibition of the proteasome and proteaphagy enhances apoptosis in FLT3‐ITD‐driven acute myeloid leukemia
Source: FEBS Open Bio. 2020 Nov 24;11(1):48–60. doi: 10.1002/2211-5463.12950 (PMC7780102; doi:10.1002/2211-5463.12950)

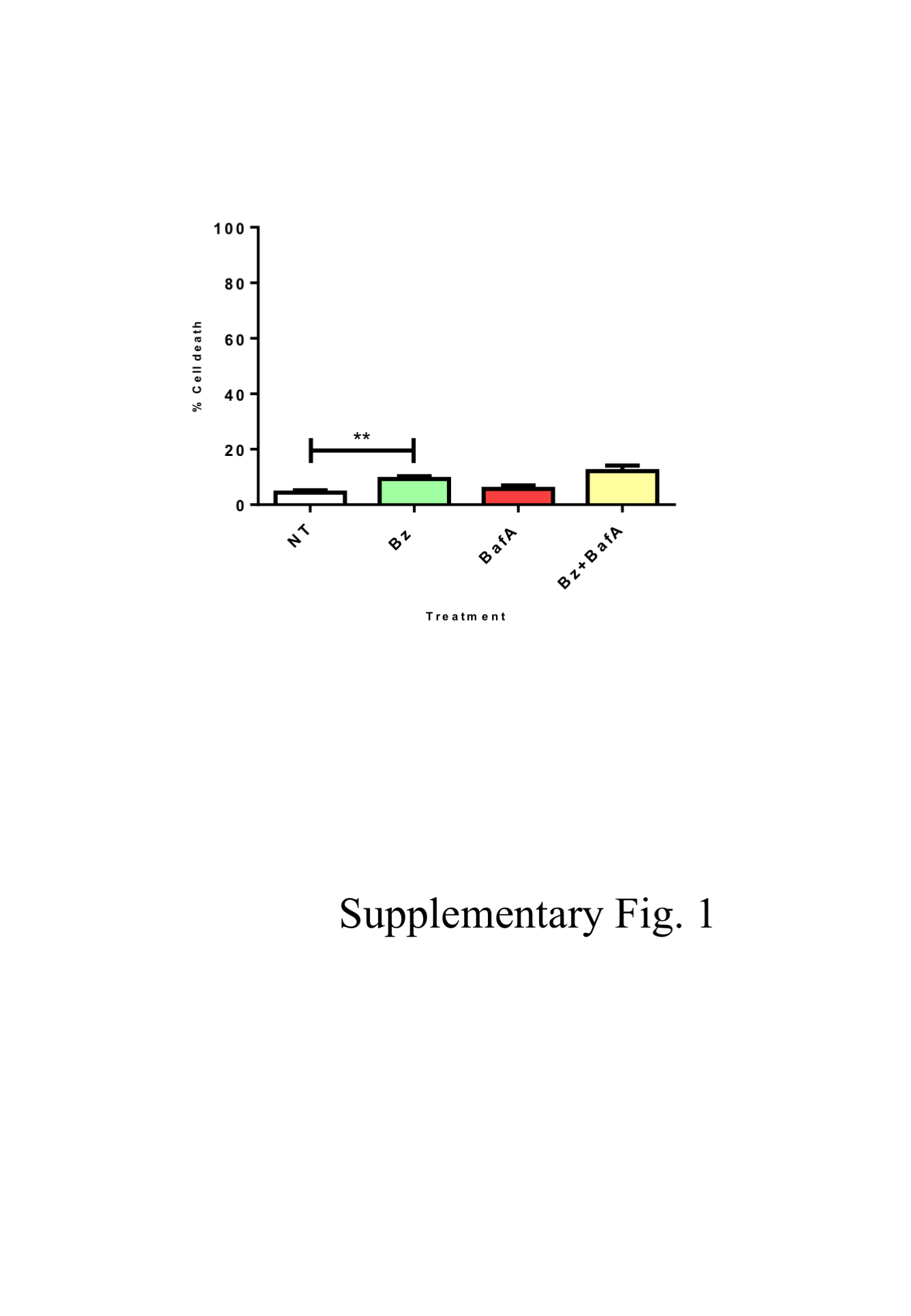

Supplement: Supplementary file 1 — Fig. S1. Cell death evaluation at 8 h. Treatments using 15 nm Bz and 10 nm BafA with fetal bovine serum 2% MOLM‐14 before annexin‐V staining and flow cytometry to validate the western blot conditions (n = 24), three biological replicates. Statistical analyses were performed using unpaired two‐tailed Student’s t‐tests with prism, version 6. *P < 0.05, **P < 0.01, ***P < 0.001 and ****P < 0.0001. Data are reported as the mean ± SEM (n = 3). [file FEB4-11-48-s001.tiff]
